# CULTURAL DIFFERENCES IN CAREGIVERS’ ATTITUDES TOWARD IN-HOSPITAL MOBILITY OF OLDER ADULTS

**DOI:** 10.1093/geroni/igae098.1793

**Published:** 2024-12-31

**Authors:** Ksenya Shulyaev, Anna Zisberg, Juliana Smichenko, Efrat Shadmi, Nurit Gur-Yash

**Affiliations:** University of Haifa, Haifa, Hefa, Israel; University of Haifa, Haifa, Hefa, Israel; University of Haifa, Haifa, Hefa, Israel; University of Haifa, Haifa, Hefa, Israel; Center of Research & Study of Aging, University of Haifa; Oranim, Academic College of Education, Israel., Haifa, HaZafon, Israel

## Abstract

Older adults are often accompanied by their family caregivers during hospitalization and their attitudes could influence care processes and outcomes. However, little is known about the cultural differences of family-caregivers attitudes. We aim to explore cultural differences in family-caregivers’ attitudes toward in-hospital mobility of hospitalized relatives. In this study participated 156 pairs of hospitalized older adults and family caregivers, included 23(15%) Israeli Arabs, 33(21%) - ex-USSR migrants and 100(64%) native, or veteran Israeli Jews. Socio-demographic, health, functional and physical status were collected at admission. Caregivers’ Attitudes Toward Mobility (ATM) were collected during their hospital visits. In all cultural groups caregivers tend to be positive toward mobility of older adults, attitudes (measured 1 for negative, 5 for positive) of the caregivers were: 3.48±.80 for Arab, 3.69±.64 for ex-USSR migrants and 3.82±.70 for Jews. Significant differences in caregivers’ attitudes between the groups were found (F(146,2)=4.02, p=.02) even after controlling for patients’ characteristics including age, gender, functional status, level of independence in ADL, severity of illness, comorbidities and previous falls. Multivariate regression analysis included all mentioned covariates demonstrated that being an Arab family-caregiver significantly predicted worse attitudes toward in-hospital mobility (β=-.214, p=.009). To conclude, cultural differences exist in attitudes of the family-caregivers regarding the mobility of their older family members. Arab caregivers tend to prefer that their relatives will be less mobile during hospitalization. Clinical practices should address cultural difference that influence mobility.

